# Supplementary material for: Does management support drive sustained agile usage? a serial mediation model and cIPMA perspective
Source: PLoS One. 2025 Feb 5;20(2):e0316538. doi: 10.1371/journal.pone.0316538 (PMC11798488; doi:10.1371/journal.pone.0316538)
Supplement: S1 Table — (DOCX) [file pone.0316538.s002.docx]

**S2 Questionnaire items**

| Item code | Questionnaire item (rated from 1 = Strongly Disagree to 5 = Strongly Agree) |
| --- | --- |
|  |  |
| MS01 | The management always makes our team members feel valued and respected. |
| MS02 | The management does not overly pressure and criticize our team members. |
| MS03 | The management helps our team set goals. |
| MS04 | The management helps our team measure progress. |
| MS05 | The management provides our team with sufficient resources for agile training. |
|  |  |
| AT01 | My agile training provides theoretical knowledge of agile practices and methodologies. |
| AT02 | My agile training helps me build confidence with internal and external stakeholders. |
| AT03 | My agile training helps in increasing motivation and willingness to experiment. |
| AT04 | My agile training helps in building shared team commitment, trust, and psychological safety. |
| AT05 | My agile training helps me identify areas for improvement. |
|  |  |
| ATLS01 | I always find new ways to do my work better. |
| ATLS02 | I like exploring new experiences in my work environment. |
|  |  |
| ATCE01 | I solve difficult challenges best when I work with my team. |
| ATCE02 | I like making my work transparent for others. |
| ATCE03 | I regularly seek feedback on my work approach from my team. |
|  |  |
| ATESG01 | I am good at organizing myself independently and setting priorities at work. |
| ATESG02 | I am open to taking on new tasks of unclear scope. |
| ATESG03 | I can independently figure out how to approach a task at work. |
|  |  |
| ATCC02 | I prefer to frequently interact with customers. |
| ATCC01 | I always think of ways to create a positive customer experience through all my work. |
|  |  |
|  |  |
| Ali01 | Our team always remains motivated and optimistic when challenges arise. |
| Ali02 | Our team always sees the achievements of team members as the team’s collective success. |
|  |  |
| Cap01 | Our team always uses members’ feedback effectively to improve performance. |
| Cap02 | Our team encourages looking for different ways to develop skills and knowledge. |
|  |  |
| Con01 | Our team always encourages a sense of team belonging. |
|  |  |
| Per01 | Our team always has effective problem-solving and decision-making approaches |
|  |  |
| Res01 | Our team always effectively uses its team members’ skills and team resources at hand. |
| Res02 | Our team continually finds ways to improve our work approach. |
| Res03 | Our team always develops team processes based on team priorities |
|  |  |
| Rob01 | Our team always focuses on shared goals over personal agendas |
| Rob02 | Our team always proactively adjusts to changes by implementing novel solutions. |
|  |  |
| Sel01 | Our team always encourages regular breaks to decrease work stress. |
|  |  |
| SAU01 | Our team always makes the best use of Agile in all software development activities. |
| SAU02 | Our team always accurately estimates the optimal number of tasks that can be accomplished within a sprint. |
| SAU03 | Our team always effectively plans and refines the detailed flow between tasks in each sprint. |
| SAU04 | Our team always implements effective solutions for blockers identified during daily stand-ups/ daily scrums. |
| SAU05 | Our team always implements the improvements discussed and agreed on during the current retrospective in the next sprint. |
| SAU06 | Our team always adjusts agile practices as needed. |
| SAU07 | Our team has deeply customized or newly created an agile metric/ practice to suit its unique needs. |
| SAU08 | Our team has clearly defined Agile team member roles which are unique to our company. |
|  |  |
