# Supplementary material for: Does management support drive sustained agile usage? a serial mediation model and cIPMA perspective
Source: PLoS One. 2025 Feb 5;20(2):e0316538. doi: 10.1371/journal.pone.0316538 (PMC11798488; doi:10.1371/journal.pone.0316538)
Supplement: Attachment — Submitted filename: Comments.docx [file pone.0316538.s003.docx]

**Abstract**

- **Clarity and Conciseness**: The abstract covers several points, but some sentences are long and complex. Breaking them into shorter, clearer sentences could enhance readability. For example, the sentence starting from "Therefore, this study aims to investigate..." could be streamlined.
- **Rewording Objectives**: Instead of listing the research aims with numbers, you might consider rephrasing them into a more fluid narrative. This will make the objectives feel more integrated into the text.
- **Reduce Repetition**: Phrases like "sustained agile usage" are repeated multiple times. You could rephrase some sentences to avoid redundancy, like using synonyms or restructuring the sentence.
- **Emphasize Key Results**: The results section could be more concise while still highlighting the core findings. Try summarizing the most significant insights in a way that is easy to grasp quickly.
- **Final Thoughts/Contributions**: The last part of the abstract mentions potential future research directions. While this is helpful, you might want to focus more on the contribution of your current research to the field. Future research directions could be reduced or briefly mentioned in the discussion section.

**Introduction**

- **Improve Flow and Coherence**: The first paragraph introduces the broader challenges in software development and agile adoption, but the transition to the discussion on *SAU* feels abrupt. You could provide a smoother transition by briefly explaining the move from agile adoption to sustainment, tying it more clearly to the initial challenges of volatility and complexity.
- **Tighten the Literature Review**: The discussion of prior studies (e.g., Senapathi and Drury-Grogan’s work) could be condensed for brevity. While it's important to highlight key contributions, ensuring that the text doesn’t become overly focused on specific studies might help maintain reader engagement.
- **Shorten Sentences for Clarity**: Several sentences are long and complex. Breaking them up can enhance clarity. For instance, *"This turbulent environment has necessitated...a shift towards agile SD (ASD)"* could be split into two sentences to make it more digestible.
- **Highlight Research Gaps More Clearly**: The section on research gaps could be more concisely summarized to focus on what’s missing in the literature. You mention gaps related to post-adoption stages of agile and under-explored human factors. It might help to present these gaps upfront before diving into the detailed literature review.
- **Use Clear Transitions to RQs**: The lead-up to the research questions could be more cohesive. Instead of stating *"Therefore, our research aims to fill the aforementioned gaps..."*, explicitly stating the importance of the identified research gaps before moving into the research questions would make it smoother.
- **Reinforce the Novelty and Contribution Early**: The novelty of using advanced techniques like IPMA, NCA, and PLS-SEM should be emphasized earlier. This can create a stronger case for why your research is distinct and valuable.
- **Structure**: Consider moving some of the detailed descriptions of analytical techniques (e.g., PLS-SEM, IPMA, NCA) to the methods section. While these are relevant, the introduction could focus more on contextualizing the study and its objectives.

**Literature review**

- **Lack of Critical Analysis**: While the literature review provides a summary of relevant studies, there is a lack of critical analysis and evaluation of these sources. It reads more like a descriptive summary rather than a critical synthesis of the key findings, gaps, and debates in the literature. The authors should incorporate more critique of the existing research, highlighting the strengths, limitations, and unresolved issues within the field.
- **Insufficient Identification of Gaps**: The review does not identify the specific gaps in the existing literature that the current study aims to address. The authors should explicitly mention what is missing or underexplored in the body of knowledge and how their study will fill these gaps. This will enhance the justification for the research.
- **Over-reliance on Older Sources**: Several of the sources cited in the review are outdated, with some being more than five years old. While classic references can be useful, there should be a stronger emphasis on recent studies to reflect the current state of research in the field. Including more up-to-date studies, particularly from the past three years, will improve the relevance of the review.
- **Lack of Theoretical Framework:** The literature review lacks a clear and cohesive theoretical framework. While several concepts are mentioned, it is not clear how they connect to form a theoretical foundation for the research. The authors should consider integrating a well-established framework or theory that will guide the development of their hypotheses and analysis.
- **Inadequate Justification for Key Variables**:

The literature review does not adequately explain why the specific variables (e.g., sustained agile usage, management support, team resilience) were selected for this study. More discussion is needed to justify the inclusion of these variables and their importance in the context of agile practices. This will help in building a stronger rationale for the research model.

**Methodology**

- **Sample Size Calculation**: The authors used a robust approach to calculate the minimum required sample size using the inverse square root method and the guidelines by Hair et al. However, it would be beneficial if the authors provided more detail on how the anticipated path coefficients (0.11-0.2) were determined. Were these values based on previous studies or preliminary data? Providing this context would strengthen the justification for the sample size calculation.
- **Sampling Approach**: The stratified multi-stage cluster sampling approach is appropriate for ensuring representation across different company sizes, but the authors could provide additional details on how the strata were determined. For example, was there any justification for the chosen employee size range (10-200 for SMEs, over 200 for large companies)? Further, clarity on how agile team members were randomly selected from within each stratum would enhance transparency.
- **Pilot Testing and Questionnaire Validation**: The authors conducted a pilot test on 45 agile software development professionals and consulted an agile expert for content validation. While this strengthens the reliability of the instrument, more details on the specific changes made after the pilot test would be useful. What kind of feedback was received, and how did it influence the final version of the questionnaire?
- **Response Exclusion**: The exclusion of 21 responses due to "suspicious response patterns" is noted. However, it would be beneficial if the authors provided more detail on the criteria used to identify these suspicious patterns. Were these cases of straight-lining or other forms of non-engaged responses? Clearly stating the exclusion criteria would help in understanding how data quality was maintained.
- **Demographic Breakdown**: The demographic breakdown of the respondents is well-presented, including gender, experience, company size, and team size. However, it may be valuable to provide a discussion on the potential impact of gender imbalance (77.75% male respondents) on the generalizability of the findings. Are there any implications of this skewed distribution for interpreting the results?

**Results**

- **Detailed Descriptions of Constructs:** While the mean scores are provided for each construct, a brief interpretation of these values in terms of their implications for the study context would add depth. Discussing the significance of the mean scores being above average could offer insights into the current state of agile practices among the study population.
- **Visual Representation:** Consider including visual representations (such as graphs or charts) alongside the descriptive statistics. This could enhance reader engagement and provide a clearer snapshot of the data distributions and correlations.
- **Measurement Model Assessment Details:** The authors reference established guidelines for evaluating construct validity but could benefit from including more details on the specific tests conducted. For instance, explicitly stating the thresholds used for reliability and validity could strengthen the rigor of the analysis.
- **Justification of Reflective Model:** While the authors state that all constructs were modeled as reflective, it might help to briefly justify this decision. A discussion of why reflective measurement was appropriate in this context, as opposed to formative measurement, would clarify the methodological choices.

**Discussion**

- The discussion section is well-presented
- Authors should expand theoretical and practical implications
- Conclusions and limitations should be separate
