# Supplementary material for: Does management support drive sustained agile usage? a serial mediation model and cIPMA perspective
Source: PLoS One. 2025 Feb 5;20(2):e0316538. doi: 10.1371/journal.pone.0316538 (PMC11798488; doi:10.1371/journal.pone.0316538)
Supplement: Attachment — Submitted filename: Response to Reviewers.docx [file pone.0316538.s004.docx]

**Editor’s Comments**

| **Editor’s Comments** | **Response to the Comment** |
| --- | --- |
| 1. Please ensure that your manuscript meets PLOS ONE's style requirements, including those for file naming. The PLOS ONE style templates can be found at <https://journals.plos.org/plosone/s/file?id=wjVg/PLOSOne_formatting_sample_main_body.pdf> and <https://journals.plos.org/plosone/s/file?id=ba62/PLOSOne_formatting_sample_title_authors_affiliations.pdf>. | Thank you very much for bringing this to our attention.  In line with the feedback, we have included a revised first page, adhering to the formal journal guidelines.  Next, we have altered the names of the supporting information files to fit the recommended format. |
| 2. In the ethics statement in the Methods, you have specified that verbal consent was obtained. Please provide additional details regarding how this consent was documented and witnessed, and state whether this was approved by the IRB. | Duly noted, thank you.  Informed verbal consent was obtained from all participants before they were provided with the questionnaire. Respondent concerns were clarified prior to participation. Participant consent was witnessed by the authors and documented in study records. The study was approved by the Sri Lanka Institute of Information Technology (SLIIT) Business School Ethics Review Committee (SLIIT/ERC/SBS/2023/17), as it was deemed appropriate for the nature of the study and minimised participant burden. |
| 3. Please include your full ethics statement in the ‘Methods’ section of your manuscript file. In your statement, please include the full name of the IRB or ethics committee who approved or waived your study, as well as whether or not you obtained informed written or verbal consent. If consent was waived for your study, please include this information in your statement as well. | Thank you for bringing this to our attention.  A separate section ‘Ethical consideration’ **(line 620 of the revised manuscript without tracked changes)** was created, in which the following was included **(line 621-627 of the revised manuscript)**.  *“To ensure ethical consent, participants were provided with detailed information about the study's objectives and the questionnaire.  All concerns were clarified prior to obtaining consent for participation. Explicit verbal consent was obtained from each respondent. Participation was entirely voluntary, and only those who explicitly consented were provided with access to the questionnaire. This consent process was witnessed by the authors. The study was conducted with the ethical approval of the Sri Lanka Institute of Information Technology (SLIIT) Business School Ethics Review Committee (SLIIT/ERC/SBS/2023/17).”* |
| 4. Please ensure that you refer to Figure 1 in your text as, if accepted, production will need this reference to link the reader to the figure. | Extremely humble apologies for the oversight.  We have included the requested as below **(line 561-562 in the revised manuscript):**  *“Fig 1 presents the proposed research framework and the hypotheses H_1_ – H_5_ of the current study.”* |

**Reviewers’ Comments**

- **Abstract**

| **Reviewers’ Comment** | **Response to the Comment** |
| --- | --- |
| 1. **Clarity and Conciseness**: The abstract covers several points, but some sentences are long and complex. Breaking them into shorter, clearer sentences could enhance readability. For example, the sentence starting from "Therefore, this study aims to investigate..." could be streamlined. | Thank you very much for bringing this to our attention.  To improve the clarity and enhance conciseness, we have modified the mentioned sentence (line 62-69 of the original manuscript) as follows by breaking it down into four smaller sentences **(line 34-40 in the revised manuscript):**  *“Therefore, this study examines if management support impacts the sustained usage of agile methodologies within software development teams. It subsequently investigates the individual and serial mediating effects of agile training, the agile mindset, and team resilience on this relationship. Additionally, it compares the importance and performance of management support, agile training, the agile mindset, and team resilience in infusing agile practices. Finally, it determines these antecedents’ necessity for the enduring success of agile application.”*  The sentence “Partial least squares structural equation modelling, importance-performance map analysis and necessary condition analysis were used as analytical methods to investigate hypotheses.” (line 70-72 of the original manuscript) was shortened and modified as below **(line 41-43 of the revised manuscript):**  *“Partial-least-squares structural equation modelling, importance-performance map analysis and necessary condition analysis were used to investigate relationships.”*  The sentence “A lack of attention to management support, agile training, agile mindset and team resilience can hinder the long-term success of agile practices” on line 60-61 of the original manuscript was reworded thus to ensure better clarity and be more direct **(line 32-33 of the revised manuscript):**  *“Human factors like management support, agile training, agile mindset, and team resilience are often neglected, hindering long-term success.”*  The sentence section “The results showed a substantial impact of management support on sustained agile usage…thresholds of all four antecedents were found to be necessary to ensure optimal sustained agile usage.” (line 72-77 of the original manuscript) was modified thus **(line 43-47 of the revised manuscript):**  *“The findings underscore the pivotal role of management support in infusing agile practices. Agile training, mindset, and team resilience emerge as critical mediators, with a strong serial mediation effect. While management support is paramount, its practical implementation falls short within teams. All four antecedents are found to be necessary for optimal agile sustainment.”* |
| 2. **Rewording Objectives**: Instead of listing the research aims with numbers, you might consider rephrasing them into a more fluid narrative. This will make the objectives feel more integrated into the text. | We sincerely appreciate your helpful advice.  This has been adjusted as required by incorporating the objectives into four separate sentences **(line 34-40 in the revised manuscript)** as shown in the response to the comment 1.  *“Therefore, this study examines if management support impacts the sustained usage of agile methodologies within software development teams. It subsequently investigates the individual and serial mediating effects of agile training, the agile mindset, and team resilience on this relationship. Additionally, it compares the importance and performance of management support, agile training, the agile mindset, and team resilience in infusing agile practices. Finally, it determines these antecedents’ necessity for the enduring success of agile application.”* |
| 3. **Reduce Repetition**: Phrases like "sustained agile usage" are repeated multiple times. You could rephrase some sentences to avoid redundancy, like using synonyms or restructuring the sentence. | We are grateful for your constructive criticism.  In response, first, the section detailing the study objectives (line 62-69 of the original manuscript) have been reworded and restructured **(line 34-40 of the revised manuscript)**, as indicated in the response to comment 1 and 2:  *“Therefore, this study examines if management support impacts the sustained usage of agile methodologies within software development teams. It subsequently investigates the individual and serial mediating effects of agile training, the agile mindset, and team resilience on this relationship. Additionally, it compares the importance and performance of management support, agile training, the agile mindset, and team resilience in infusing agile practices. Finally, it determines these antecedents’ necessity for the enduring success of agile application.”*  In addition, the results section (line 72-77 in original manuscript) was reworded thus **(line 43-47 in the revised manuscript)** as indicated in the response to comment 1:  “*The findings underscore the pivotal role of management support in infusing agile practices. Agile training, mindset, and team resilience emerge as critical mediators, with a strong serial mediation effect. While management support is paramount, its practical implementation falls short within teams. All four antecedents are found to be necessary for optimal agile sustainment.*” |
| 4. **Emphasize Key Results**: The results section could be more concise while still highlighting the core findings. Try summarizing the most significant insights in a way that is easy to grasp quickly. | We thank you for your insightful observations.  In response to your helpful suggestions, the results section (line 72-77 of the original manuscript) has been rewritten and summarised **(line 43-47 in the revised manuscript)** as indicated in the response to comment 1 and 3:  “*The findings underscore the pivotal role of management support in infusing agile practices. Agile training, mindset, and team resilience emerge as critical mediators, with a strong serial mediation effect. While management support is paramount, its practical implementation falls short within teams. All four antecedents are found to be necessary for optimal agile sustainment.*” |
| 5. **Final Thoughts/Contributions**: The last part of the abstract mentions potential future research directions. While this is helpful, you might want to focus more on the contribution of your current research to the field. Future research directions could be reduced or briefly mentioned in the discussion section. | Thank you very much for your erudite commentary.  In response, the discussion section, starting line 77-78 in the original manuscript has been expanded thus to focus more on the study’s contribution **(line 47-54 in the revised manuscript)**:  “*Thus, this study significantly advances theoretical understanding by introducing a serial mediation model that elucidates their mechanisms in impacting agile infusion. It extends prior organisational-level findings to the team-level. The study's quantitative verification of qualitative findings strengthens their generalisability to a broader spectrum of teams. It pioneers in expounding the constructs’ relative importance, performance and necessity, to offer actionable insights for agile practitioners. Finally, it provides methodological guidance to apply importance performance map analysis and necessary condition analysis in agile software development research.”* |

- **Introduction**

| **Reviewers’ Comment** | **Response to the Comment** |
| --- | --- |
| 1. **Improve Flow and Coherence**: The first paragraph introduces the broader challenges in software development and agile adoption, but the transition to the discussion on SAU feels abrupt. You could provide a smoother transition by briefly explaining the move from agile adoption to sustainment, tying it more clearly to the initial challenges of volatility and complexity. | Thank you very much for taking the time to share your constructive criticism.  To address this concern, we have included the following sentences **(line 71-74 in the revised manuscript):**  “*Nonetheless, an ASD team’s journey towards realising the benefits of agile extends beyond the mere adoption of agile practices. While agile methodologies offer a promising approach to navigating the volatile, uncertain, complex, and ambiguous landscape of software development, their long-term success hinges on their sustained or enduring usage [15,17–19].*”  The following sentence “Nonetheless, to effectively ensure the complete realisation of the benefits offered by agile methodologies, it is imperative that it is not only adopted but also sustained within development teams.” (line 96-98 of original manuscript) was restructured to integrate it further into the narrative. The modified sentence reads as follows **(line 75-76 in the revised manuscript)**:  *“In other words, to effectively ensure the complete realisation of the benefits of agile methodologies, they should not only be adopted but also sustained within ASD teams [15,17–19].* |
| 2. **Tighten the Literature Review**: The discussion of prior studies (e.g., Senapathi and Drury-Grogan’s work) could be condensed for brevity. While it's important to highlight key contributions, ensuring that the text doesn’t become overly focused on specific studies might help maintain reader engagement. | We appreciate your invaluable input.  To address this concern, we have condensed and restructured the discussion on prior work (line 108-127 in the original manuscript) thus **(line 97-114 in the revised manuscript)** to incorporate different studies, while providing more details on the relationships between variables. This revision ensures a smooth flow, with less emphasis on specific studies.  *“Recognising their pivotal importance, scholars have endeavoured to investigate the sociological antecedents of SAU. Prior studies specifically highlight the roles of management support (MS), agile training (AT), and the agile mindset (AM) as critical success factors for organisational sustainment of agile [15,25,30]. Strong MS is crucial for providing necessary resources, fostering a supportive culture, and encouraging continuous improvement. AT was found vital to impart technical/business knowledge and facilitate effective team dynamics. An AM, characterised by a willingness to improve, collaborate, take initiative, and focus on customer needs, was also deemed essential. Moreover, research emphasises the importance of team resilience (TR) in SD team success [31]. TR is a team's ability to withstand, adapt to and recover from adversity, with minimum impairment to its functioning [32,33]. SAU necessitates continually evolving team practices, while maintaining optimal performance amidst unforeseen challenges. In such circumstances, TR emerges as a determinant between team success and failure [34–37]. However, studies show that MS can directly impact both AT and AM [38]. Effective AT, in turn, equips team members with the skills and knowledge to adopt an agile mindset and implement agile practices. Furthermore, MS, AT and AM can contribute to TR, by helping equip teams with the necessary skills and mindset [29]. Thus, the current study contends that MS is a key initial step in sustaining agile practices, and that MS, AT, AM, and TR are interconnected factors that exert individual and serial impacts on SAU.”*  The section “While Senapathi and Drury-Grogan’s [15] study is monumental in outlining MS, AT and AM as…past study also focused on the critical success factors on an organisational level.” on page 156-159 on the original manuscript was removed and rewritten, without emphasis on the individual study **(line 140-143 of the revised manuscript).**  *“While MS, AT, and AM were qualitatively introduced as critical success factors of SAU, quantitative research is needed to verify these findings. Furthermore, as extant studies address the impact of these factors on SAU at the organisational level, failing to probe the factors at team level constitutes a critical oversight.”* |
| 3. **Shorten Sentences for Clarity**: Several sentences are long and complex. *Breaking them up can enhance clarity. For instance, "This turbulent environment has necessitated...a shift towards agile SD (ASD)"* could be split into two sentences to make it more digestible. | This comment is duly acknowledged. To address the same, the following changes have been made.  The first sentence of the introduction (line 84-87 of the original manuscript) has been split into two to enhance readability and clarity **(line 58-61 in the revised manuscript).**  “*The contemporary landscape of software development (SD) is fraught with rising volatility, uncertainty, complexity, and ambiguity [1]. This is largely triggered by the accelerating pace of technological innovation, convoluted client demands, hyper-competitiveness and the escalating pressure to deliver greater value within stiffer deadlines [2–6].”*  Furthermore, the second sentence (line 87-89 in the original manuscript), has also been split as suggested **(line 61-63 in the revised manuscript).**  “*Such a turbulent environment has necessitated that SD teams undergo a fundamental reorientation of SD practices. Thus, this has impelled a shift towards agile SD (ASD) [7–9].”*  The sentence “A growing body of research has consistently highlighted…satisfaction [12–15], which ultimately reinforces competitive advantage.” (line 90-93 of the original manuscript) has been broken down thus **(line 65-68 of the revised manuscript):**  *A growing body of research has consistently highlighted the substantial benefits of ASD, such as enhanced efficiency, less time to market, improved quality and greater customer satisfaction [12–15]. Hence, efficient agile usage ultimately reinforces competitive advantage.”*  The sentence “Yet scholars such as Gregory et al. [20] and Wang et al. [21] point out that ASD teams, having already evolved past merely adopting these methodologies, are currently facing immense challenges in this regard.” on line 100-102 of the original manuscript has been shortened thus **(line 79-81 of the revised manuscript):**  *“Yet scholars such as Gregory et al. [20] and Wang et al. [21] point out that ASD teams are currently facing immense challenges in this regard.”*  The sentence on line 104-107 in the original manuscript has been condensed thus **(line 82-84 in the revised manuscript):**  “*Such an understanding will enable optimal resource allocation and stimulate effective strategy development in the pursuit of SAU within ASD teams*.”  The sentence on line 108-110 in the original manuscript “It has been steadily documented that the success of agile methodologies is fundamentally rooted in and critically contingent upon human factors [6,11,22-24, despite many organisations and teams paying minimal attention to the same [11,32,33].” was broken into two **(line 93-96 in the revised manuscript)** to emphasise its meaning further:  *“Hence, it is evident that the efficacy of agile methodologies within teams is critically contingent upon its human factors and their interactions [6,11,23–27]. Despite this, their neglect by both scholars and practitioners [11,28,29] exacerbates challenges in sustaining agile practices within teams.”*  The sentence “In addition, researchers would largely benefit from the use of advanced techniques such as…thus helping prioritise areas for improvement [41].” on line 135-139 of the original manuscript was broken down thus to further emphasise the specific importance of IPMA **(line 120-125 of the revised manuscript)**:  *“In addition, ASD researchers and teams would largely benefit from the use of importance-performance map analysis (IPMA), that complements PLS-SEM by adding practical relevance to findings. IPMA visually contrasts the identified factors’ (MS, AT, AM and TR) importance in achieving SAU, with their current performance levels within ASD teams. By doing so, it helps pinpoint the most critical factors that require prioritisation [41], allowing effective resource allocation.”*  The sentence “This forms a critical knowledge gap…aimed at furthering SAU.” on line 162-165 in the original manuscript has been shortened as below, placing more emphasis on the immediate outcome SAU **(line 147-148 in the revised manuscript).**  “*Thus, these limitations form a critical knowledge gap that will severely impede the development of effective interventions to sustain agile practices.*”  The single sentence “The study incorporates three individual mediation effects…how the interplay of MS, AT, AM and TR ultimately lead to the development of SAU.” (line 182-184 in the original manuscript) has been split into two for a more fluid reader experience. The revised sentences read thus **(line 183-185 in the revised manuscript):**  *“The study incorporates three individual mediation effects and a serial mediating mechanism into a single unified framework. Thus, it provides a broad and complex comprehension of how the interplay of MS, AT, AM and TR ultimately lead to the development of SAU.”* |
| 4. **Highlight Research Gaps More Clearly**: The section on research gaps could be more concisely summarized to focus on what’s missing in the literature. You mention gaps related to post-adoption stages of agile and under-explored human factors. It might help to present these gaps upfront before diving into the detailed literature review. | Your feedback is much appreciated and extremely helpful. We understand that it would be beneficial to the reader if the gaps were presented upfront, to ensure clarity and focus.  In line with this suggestion, the sentence “Furthermore, human factors…remain under-explored within the domain of SAU.” was relocated from line 187-188 in the original manuscript to **line 137-139 in the revised manuscript** to provide an upfront and more detailed account of the gap of exploring human factors, putting the current study into context more clearly.  The section detailing the research gaps (line 153-168 of the original manuscript) was rewritten to explicitly state the same **(line 132-148 of the revised manuscript).**  *“However, despite the pressing need for novel research on SAU on these fronts, the existing body of research is significantly plagued by numerous limitations. First, no known study has incorporated the added perspectives of PLS-SEM, IPMA and NCA (cIPMA) to the exploration of SAU, significantly curtailing the practical utility of existing knowledge. Next, the majority of studies focus on the adoption stage of ASD, neglecting the equally, if not more, important area of post-adoptive usage [20,21,26]. Furthermore, human factors accounting for a large portion of the challenges faced by teams remain under-explored within the already sparse domain of SAU. While MS, AT, and AM were qualitatively introduced as critical success factors of SAU, quantitative research is needed to verify these findings. Furthermore, as extant studies address the impact of these factors on SAU at the organisational level, failing to probe the factors at team level constitutes a critical oversight. Moreover, despite research conceding that TR is crucial in the context of ASD, very limited investigation of its impact on agile infusion exists. Additionally, the mechanisms through which MS, AT, AM, and TR influence SAU remain poorly understood. This is because limited research has explored the role of AT, AM, and TR as potential individual and serial mediators in the relationship between MS and SAU. Thus, these limitations form a critical knowledge gap that will severely impede the development of effective interventions to sustain agile practices.”* |
| 5. **Use Clear Transitions to RQs**: The lead-up to the research questions could be more cohesive. Instead of stating *"*Therefore, our research aims to fill the aforementioned gaps...", explicitly stating the importance of the identified research gaps before moving into the research questions would make it smoother. | Thank you very much for the insightful comment. This would indeed be helpful in maintaining reader engagement.  To emphasise the importance and critical nature of fulfilling the research gaps prior to addressing how our study attempts to do the same, the following sentence was added **(line 149-153 in the revised manuscript).**  *“The failure to develop effective interventions towards sustaining agile within the already challenging landscape of ASD would prove largely detrimental for teams. The incomplete application and benefit realisation of agile methodologies resulting from such knowledge scarcity could potentially lower team agility, decrease productivity and innovation, ultimately eroding competitive advantage.”*  Furthermore, the sentence on line 167-168 of the original manuscript “Therefore, our research aims to fill the aforementioned gaps through answering five research questions.” was expanded to place it more smoothly into the paragraph’s context. This revision reads thus **(line 153-155 in the revised manuscript):**  “*Therefore, to bridge these critical knowledge gaps and ensure the continued success of agile methodologies, our research aims to address the following five research questions*.” |
| 6. **Reinforce the Novelty and Contribution Early**: The novelty of using advanced techniques like IPMA, NCA, and PLS-SEM should be emphasized earlier. This can create a stronger case for why your research is distinct and valuable. | We appreciate the suggestion to emphasise the novelty of our research early. As pointed out, doing so will definitely strengthen the argument for the study’s unique contribution to the field.  To incorporate this feedback, we restructured and rewrote the paragraph detailing the study’s contributions (line 176-194 of the original manuscript). In the revised version, the contribution of the cIPMA perspective was moved from the latter part of the paragraph to the earlier part. The contributions of the research were detailed under methodological advancements, practical insights and theoretical advancements within ASD research. All three sections emphasise the contribution of the IPMA and NCA (cIPMA results).  The revised section is given thus **(line 167-189 of the revised manuscript)**:  *“This research offers a groundbreaking contribution to the broader field of ASD in three key ways.*  *First, it offers a significant methodological advancement of ASD research by pioneering the utilisation of cIPMA in the field. The combined application of PLS-SEM, IPMA and NCA allows for a more rigorous and comprehensive analysis of the complex relationships. This novel approach sets a new standard for ASD research and practice. By doing so, it provides a referential guide for future studies looking to examine similar phenomena within the arena.*  *Second, the study provides significant actionable insights for agile practitioners (both team members and managers) by incorporating the findings of PLS-SEM, IPMA and NCA (cIPMA). The incorporation of IPMA and NCA empowers teams to identify and address performance gaps, allocate resources effectively, mitigate risks and streamline decision making. The theoretical framework developed in the study can be used to design and implement targeted interventions to improve teams’ SAU.*  *Third, the study provides a significant theoretical advancement within the domain of SAU. It pioneers in providing quantitative evidence for the necessity and impact of four critical success factors of SAU at the team level. The study incorporates three individual mediation effects and a serial mediating mechanism into a single unified framework. Thus, it provides a broad and complex comprehension of how the interplay of the necessary factors MS, AT, AM and TR ultimately lead to SAU. Furthermore, it makes a valuable addition to the literature on post-adoptive agile usage which is an extremely under-researched yet important area [15,21,26]. This is crucial considering that teams face significant challenges in this regard. It also makes huge strides in exploring human or sociological factors affecting SAU, an underexplored area within the wider domain of ASD.”* |
| 7. **Structure**: Consider moving some of the detailed descriptions of analytical techniques (e.g., PLS-SEM, IPMA, NCA) to the methods section. While these are relevant, the introduction could focus more on contextualizing the study and its objectives. – | We would like to express our sincere gratitude for your valuable feedback. Your observation is indeed correct and helpful to improve readability.  Note that the following section (line 132-135 in the original manuscript “This technique is popular for its minimal distributional assumptions…generalise research finding to the actual world, to facilitate beneficial practitioner implications [35].” included in the description of PLS-SEM in the introduction has been removed in the revised manuscript. This is because the ‘methods’ section includes a comprehensive description of the technique and covers the removed technical details.  The sentence “While PLS-SEM and IPMA are valuable tools… and are ‘sufficient but not necessary’ to ensure the result.” on line 141-143 in the original manuscript has been broken into two sentences thus:  “*While PLS-SEM and IPMA are valuable tools, they operate predominantly on a sufficiency additive logic of causality. This focuses on assessing whether factors have a strong impact on an outcome and are ‘sufficient but not necessary’ to ensure the result*.”  Next, the modified paragraph section was moved to **line 761-763 in the methods section of the revised manuscript.**  Similarly, the sentence “Solely relying on these techniques can…of the causal relationships involved.” (line 146-147 of the original manuscript) was also moved to **line 764-765 in the revised manuscript**.  The resulting paragraph section (adjusted and modified to integrate it) in the methods section reads thus **(line 761-767 in the revised manuscript):**  “*While PLS-SEM and IPMA are valuable tools, they operate predominantly on a sufficiency additive logic of causality, which focuses on assessing whether factors have a strong impact on an outcome and are ‘sufficient but not necessary’ to ensure the result. This perspective, although essential, might be incomplete. Solely relying on these techniques can therefore obscure a more nuanced understanding of the causal relationships involved. To address this, a necessary condition analysis (NCA) [43,44] was undertaken in order to test hypotheses H_6_ – H_9_*.”  Additionally, the sentence “This constitutes the necessity logic of causality.” (line 149-150 in the original manuscript) was removed to focus more on contextualising the current study within the introduction. This information was already present in the ‘methods’ section.  The two paragraphs detailing the techniques (line 128-140 and line 141-152 of the original manuscript) were combined into one and rewritten. The new section makes an effort to address how the techniques are relevant in addressing how MS, AM, AT and TR impact SAU, without too many technical details. The rewritten section reads thus **(line 115-131 of the revised manuscript):**  *“Given the importance of agile infusion for combatting the challenges of the ASD landscape, it is crucial to probe how the key factors of MS, AT, AM and TR impact SAU from different logical perspectives. Practical applicability being paramount, researchers can harness combinations of advanced analytical techniques to gain actionable insights. A commonly used and widely potent technique for modelling complex relationships is partial least squares structural equation modelling [39,40]. In addition, ASD researchers and teams would largely benefit from the use of importance-performance map analysis (IPMA), that complements PLS-SEM by adding a layer of practical relevance to the findings.  IPMA visually contrasts the identified factors’ (MS, AT, AM and TR) importance in achieving SAU, with their current performance levels within ASD teams. By doing so, it helps pinpoint the most critical factors that require prioritisation [41], allowing effective resource allocation. However, a construct, albeit exhibiting low impact based on PLS-SEM and IPMA results, may still be a necessary condition for achieving optimal SAU [42]. A necessary condition analysis (NCA) [43–45] adds a novel perspective to the current study’s results, by assessing if a certain level of MS, AT, AM or TR is necessary for SAU to truly manifest [42]. Thus, this novel approach combining PLS-SEM, IPMA and NCA (styled cIPMA) [42] significantly enhances the rigor of the current study. Such nuanced and profound understanding of SAU bolsters its practical implications for ASD teams.”* |

- **Literature Review**

| **Reviewers’ Comment** | **Response to the Comment** |
| --- | --- |
| 1. **Lack of Critical Analysis**: While the literature review provides a summary of relevant studies, there is a lack of critical analysis and evaluation of these sources. It reads more like a descriptive summary rather than a critical synthesis of the key findings, gaps, and debates in the literature. The authors should incorporate more critique of the existing research, highlighting the strengths, limitations, and unresolved issues within the field. | Thank you very much for the constructive criticism.  To address this comment, as well as the rest of the comments on the literature review section, we have reorganised the structure of the literature review.  First the section ‘The concept of sustained agile usage’ **(line 198 of the revised manuscript)** was modified to include a critical analysis of current literature. This section details the post-adoptive usage of agile, its importance and the gaps pertaining to the lack of research focused on SAU. Furthermore, it explains the gap of the lack of investigation of SAU at the team level. It then mentions the gap of quantitative verification of SAU.  Following are excerpts from the section.  **Line 199-212 of the revised manuscript:**  *“Agile methodologies are a response to a critical need for SD teams to be flexible, adaptable and continually competitive in the midst of numerous challenges. [7–11]. However, while the initial adoption of agile methodologies is a critical first step, this is insufficient to ensure the long-term realisation of agile benefits [15,19,21,26,46,47]. Bhattacharjee [17] argues that to make the best use of any innovation or methodology, it needs to be used enduringly and persistently while being adapted to suit the operating context. It is therefore implied that contextual optimisation of the practice is crucial. Without such sustained use, the application of agile methodologies within teams would remain primitive, highlighting the importance of the concept of SAU. However, teams often encounter obstacles in their efforts towards the same [19,21,30,46], constituting a significant business problem. Hence, making a clear distinction between adoption and sustained usage of agile practices is essential to comprehensively assess the implications of the same. Thus, the current study aims to make a valuable contribution to the understanding of ASD by investigating the post-adoption or sustained stage of agile methodologies.*  **Line 251-279 of the revised manuscript:**  *“It is evident that the arena of SAU presents a critical area of investigation for practitioners and academics alike, due to the need for the realisation of benefits offered by agile methods. Nonetheless, the scrutinisation of the extant literature on ASD reveals major limitations in their applicability. First, the underrepresentation of recent research focused on the sustained usage phase of agile methodologies is especially pronounced. While numerous studies have explored the initial adoption of agile practices [38,50–53], agile success [54] and agile team outcomes [55], there is a dearth of research into sustained usage of agile practices. In other words, research tends to address either the inputs or outputs of agile implementation, without investigating the journey of agile practice evolution. This oversight limits the understanding of true long-term agile optimisation. Furthermore, studies’ lack of clear conceptual distinction between usage and sustained usage of agile implementation [18,26] further exacerbates the research gap. Many studies conflate these two stages, leading to vague conceptualisations and a failure to recognise the unique challenges and opportunities associated with each phase. Despite seminal work providing a theoretical foundation for understanding the post-adoption phase [15,25], subsequent research has largely ignored this conceptualisation [26,54]. This has resulted in a fragmented and inconsistent body of knowledge. Thus, the current study aims to advance the field by adhering to a rigorous conceptualisation of SAU, addressing the limitations of previous research.*  *The overreliance on case study and qualitative methodologies in the existing literature [26,30,56] also limits understanding of SAU, as well as practical applicability and generalisation. This methodological limitation, compounded by the lack of quantitative verification of the studies, hinders robust theoretical advancements and practical guidelines [15,19,20,28,46]. Moreover, the perspective of the team, the primary work unit in ASD, is largely neglected in the already scant research domain. Multiple scholars [30] examine the concept on the organisational level [15,19,26,30,46]. Thus, the resulting lack of unified and theoretically sound knowledge on effectively utilising agile would potentially lead to lost competitive advantage and reduced profitability of ASD teams. Such a background critically underscores the need for deep investigation into SAU. Thus, the current study will quantitatively investigate the post-adoptive stage of agile on the team level.”*  Next, a section named *‘The human perspective of agile teams’* was added **(line 280 of the revised manuscript).**  This section emphasises the importance and prominence of human factors in SAU using the perspective of the **Socio-technical Theory**. Then, it will explain how the gap of insufficient focus on human factors would lead to challenges in agile infusion. Next, it will provide empirical evidence for the importance of the factors of MS, AT, AM and TR in the context of SAU. Then it will address how the factors interact to dynamically impact SAU, using the **Input-Mediator-Output-Input model of teams.** Then it will detail the lack of a cohesive framework that addresses the mechanisms of how these affect SAU.  Following are excerpts from the section.  **Line 281-360 of the revised manuscript:**  *“The socio-technical theory of information system research [22,59] posits that any system, such as an agile team [60], comprises two interconnected subsystems: the social subsystem and the technical subsystem. The social subsystem encompasses the human elements, including team members, management, their interactions, behaviours, attitudes, team dynamics and cultural norms. The technical subsystem, on the other hand, consists of the tools, technologies, practices and processes employed within the system [61]. The two subsystems show a substantial reciprocal influence on each other, ultimately shaping the nature and direction of the holistic system [62]. However, from a sociotechnical perspective, the technical subsystem serves as a tool that empowers individuals and teams, rather than dictating their actions. This perspective emphasises humans as the active agents in shaping the technical component [63]. Therefore, successful change within a socio-technical system, such as the optimisation of agile, relies primarily on its social element [64]. Thus, this theory provides a strong theoretical foundation for assessing the human factors that influence agile adoption and sustainment.*  *Empirical evidence recognises the fundamental role of human factors in the success of agile practices [11,15,19,28,57,59,64,65]. The ‘Agile Manifesto’ [24] emphasises the importance of human factors over technical processes. As such, it is human-related challenges that pose significant barriers to the successful implementation and sustainment of a team’s agile practices [23,66]. Senapathi and Strode [30] elaborate that the path to agile sustainment is riddled with challenges related to the people involved and their lack of readiness to embrace agile values. They cite the lack of agile mindsets, leadership support, and unconducive team dynamics as factors. This aligns with Denning's [16] assertion that true agility requires a cultural shift, going beyond the mere adoption of practices to embrace agile values and behaviours. To be precise, teams are required to ‘be agile, rather than do agile’. Therefore, insufficient scholarly and practitioner focus on such human factors significantly inhibit the optimisation of agile methodologies, However, despite the importance of human factors within the context, research providing strong evidence for the role of such factors in SAU is still in infancy. Hence, applying a socio-technical lens to the study of SAU, this research aims to investigate how specific human factors contribute to successful agile sustainment.*  *In their seminal work, Senapathi and Drury-Rogan [15] qualitatively introduced and refined a foundational framework for the critical success factors of organisational SAU. In this model, they underlined the pivotal roles of sociological influences. MS, by way of actively facilitating the implementation of agile through providing resources, training, creating a supportive culture and empowering teams, was found to be instrumental in fostering SAU. Furthermore, they elucidated the vital role of the AM. They asserted that its inherent characteristics such as the enthusiasm to learn, willingness to collaborate, proactive and independent initiative, and customer-centricity were central to ensuring agile assimilation in teams. Likewise, the importance of AT and mentoring of team members to this end was demonstrated to be significant. It was recognised that the imparting of technical and business knowledge pertaining to agile methodologies while facilitating agile team dynamics through such training is crucial for SAU. However, the study failed to address the perspective of the ASD team, instead investigating the relationships on the organisational level. Subsequent research has failed to build upon this seminal study with recent empirical verifications. Furthermore, limited quantitative analysis has been carried out with regards to the impacts of the variables on SAU. Thus, the importance of the MS, AT and AM for team level SAU remain unresolved and requires further empirical investigation.*  *Moreover, researchers such as Cheng et al. [31] emphasise the importance of the ability of SD teams to effectively adapt to change, overcome challenges, and maintain their performance in order to achieve agile success. This coincides with the concept of TR which denotes the ability of teams to weather change and adversity with minimal functional detriment [29,55,64]. Since SAU requires ongoing change, it is reasonable to posit that TR is a prominent factor in the success of the same. However, the lack of prior research on TR in the context of SAU presents a significant opportunity to explore its impact on agile infusion. Accordingly, given their strong theoretical and empirical foundation in ASD literature, this study selected MS, AT, AM, and TR as key human factors to investigate their impact on SAU.*  *In accordance with the sociotechnical perspective [22,58], it can be argued that the human aspects within the system of an ASD team interact with each other. These interactions thereby impact the technical subsystem through critical dependencies. The Input-Mediator-Output-Input (IMOI) model of teams [67] provides a theoretical foundation to understand how these effects unfold. It illuminates that inputs, such as management activities, team training and team member attitudes, trigger the development of team processes and emergent states [27,55–57,68,69], such as team resilience. These processes and emergent states, in turn, gives rise to desired team outcomes, such as sustained agile usage in this context. It additionally elaborates that outcomes are further propelled by interactions between inputs, emergent states, and between inputs and emergent states. Thus, it is reasonable to posit that the factors of MS, AT, AM and TR impact SAU through dynamic interactions that have a serial impact on the same. However, a holistic investigation of these factors within a single study on agile infusion is notably absent from the literature. Due to this, a cohesive framework that addresses the mechanisms through which these factors dynamically impact SAU is still lacking.*  *The existence of the discussed research gaps has significant detrimental effects on both academic and practical spheres. The lack of knowledge hinders the ability to draw definitive conclusions and develop evidence-based recommendations for teams in different contexts. Without a clear understanding of these mechanisms, teams may struggle to effectively implement and sustain agile practices. This underscores the need for further investigation into the factors that contribute to SAU and the development of practical strategies to support teams in their agile journey. By addressing the aforementioned limitations, this study aims to provide a more comprehensive and actionable framework towards understanding mechanisms through which human factors drive sustained agile success.*  *The following sections will further expound on the relevance and interplay of the factors of MS, AT, AM and TR in the context of SAU. It will then detail the formulation of the study’s hypotheses.”*  Next, the sections detailing the individual roles of variables will provide justification for hypothesis development of the research model.  In the section “Management support and sustained agile usage” **(line 361 of the revised manuscript)** the following was added and modified.  **Line 377-379 of the revised manuscript:**  *“Given the significant role of the construct in providing resources, training, and a supportive culture, this study posits that MS is a critical foundational step in establishing and sustaining agile practices within teams.”*  **Line 379-383 of the revised manuscript:**  *“However, Senapathi and Srinivasan [18] were unable to quantitatively establish a statistically significant relationship between MS and agile usage from a sample of 114 agile practitioners. While this study provides a useful starting point, a larger sample size would be necessary to draw more definitive conclusions. Furthermore, while the study examined usage of practices, it did not address the nuances of SAU.”*  In the section “The individual mediating role of agile training in the impact of management support on sustained agile usage**” (line 387-388 of the revised manuscript)**, the following was added and modified.  **Line 406-409 of the revised manuscript:**  *“However, extant studies often overlook the comprehensive impact of AT on SAU, focusing primarily on how coaches help maintain practices. This study therefore aims to incorporate the various ways in which team members can access AT, beyond the traditional role of the agile coach.”*  In the section “The individual mediating role of agile mindset in the impact of management support on sustained agile usage” **(line 420-421 of the revised manuscript)**, the following was added and modified.  **Line 438 of the revised manuscript:**  *“Nevertheless, these studies show limited quantitative evidence.”*  **Line 455-456 of the revised manuscript:**  *“Furthermore, prior studies lack a strong theoretical conceptualisation of AM when examining its impact on SAU.”*  In the section “The individual mediating role of team resilience in the impact of management support on sustained agile usage” **(line 466-467 of the revised manuscript)**, the following was added and modified.  **Line 488-489 of the revised manuscript:**  *“However, the impact of TR on the specific outcome of SAU has not been probed in prior research.”*  **Line 503-504 of the revised manuscript:**  *“These findings, however, remain quantitatively unverified.”*  In the section “The serial mediation role of agile training, agile mindset and team resilience in the impact of management support and on sustained agile usage” **(line 509-510 of the revised manuscript)**, the following was added and modified.  **Line 524-525 of the revised manuscript:**  *“However, this serial effect has not been tested empirically, thus risking overlooking the nuanced and complex relationships between the variables.”*  In the section “Necessary conditions of sustained agile usage” (**line 529 of the revised manuscript)**, the following was added and modified.  **Line 552-554 of the revised manuscript:**  *“Such a limitation hinders a comprehensive understanding of what factors are indispensable in the ASD context.”* |
| 2. **Insufficient Identification of Gaps**: The review does not identify the specific gaps in the existing literature that the current study aims to address. The authors should explicitly mention what is missing or underexplored in the body of knowledge and how their study will fill these gaps. This will enhance the justification for the research. | We sincerely apologise for the failure to explicitly incorporate the gaps into the discussion.  Therefore, we have revised the literature review as explained above to explicitly state gaps and how the current study plans to address them.  Please note that the concern was addressed in the response to the comment above.  In the section ‘The concept of sustained agile usage’, the following was included.  **Line 207-212 of the revised manuscript:**  *“However, teams often encounter obstacles in their efforts towards the same [19,21,30,46], constituting a significant business problem. Hence, making a clear distinction between adoption and sustained usage of agile practices is essential to comprehensively assess the implications of the same. Thus, the current study aims to make a valuable contribution to the understanding of ASD by investigating the post-adoption or sustained stage of agile methodologies.”*  **Line 253-279 of the revised manuscript:**  *“Nonetheless, the scrutinisation of the extant literature on ASD reveals major limitations in their applicability. First, the underrepresentation of recent research focused on the sustained usage phase of agile methodologies is especially pronounced. While numerous studies have explored the initial adoption of agile practices [38,50–53], agile success [54] and agile team outcomes [55,56], there is a dearth of research into sustained usage of agile practices. In other words, research tends to address either the inputs or outputs of agile implementation, without investigating the journey of agile practice evolution. This oversight limits the understanding of true long-term agile optimisation. Furthermore, studies’ lack of clear conceptual distinction between usage and sustained usage of agile implementation [18,26,57] further exacerbates the research gap. Many studies conflate these two stages, leading to vague conceptualisations and a failure to recognise the unique challenges and opportunities associated with each phase. Despite seminal work providing a theoretical foundation for understanding the post-adoption phase [15,25], subsequent research has largely ignored this conceptualisation [26,54]. This has resulted in a fragmented and inconsistent body of knowledge. Thus, the current study aims to advance the field by adhering to a rigorous conceptualisation of SAU, addressing the limitations of previous research.*  *The overreliance on case study and qualitative methodologies in the existing literature [26,30,58] also limits understanding of SAU, as well as practical applicability and generalisation. This methodological limitation, compounded by the lack of quantitative verification of the studies, hinders robust theoretical advancements and practical guidelines. Moreover, the perspective of the team, the primary work unit in ASD, is largely neglected in the already scant research domain. Multiple scholars examine the concept on the organisational level [15,19,26,30,46]. Thus, the resulting lack of unified and theoretically sound knowledge on effectively utilising agile would potentially lead to lost competitive advantage and reduced profitability of ASD teams. Such a background critically underscores the need for deep investigation into SAU. Thus, the current study will quantitatively investigate the post-adoptive stage of agile on the team level.”*  In the section ‘The human perspective of agile teams’, **(line 280 of the revised manuscript)** the following was added.  **Line 305-308 of the revised manuscript:**  *“However, despite the importance of human factors within the context, research providing strong evidence for the role of such factors in SAU is still in infancy. Hence, applying a socio-technical lens to the study of SAU, this research aims to investigate how specific human factors contribute to successful agile sustainment.”*  **Line 320-325 of the revised manuscript:**  *“However, the study failed to address the perspective of the ASD team, instead investigating the relationships on the organisational level. Besides, subsequent research has failed to build upon this seminal study with recent empirical verifications. Furthermore, limited quantitative analysis has been carried out with regards to the impacts of the variables on SAU. Thus, the importance of the MS, AT and AM for team level SAU remain unresolved and requires further empirical investigation.”*  **Line 331-334 of the revised manuscript:**  *“However, the lack of prior research on TR in the context of SAU presents a significant opportunity to explore its impact on agile infusion. Accordingly, given their strong theoretical and empirical foundation in ASD literature, this study selected MS, AT, AM, and TR as key human factors to investigate their impact on SAU.”*  **Line 346-348 of the revised manuscript:**  *“However, a holistic investigation of these factors within a single study on agile infusion is notably absent from the literature. Due to this, a cohesive framework that addresses the mechanisms through which these factors dynamically impact SAU is still lacking.”*  **Line 349-357 of the revised manuscript:**  *“The existence of the discussed research gaps has significant detrimental effects on both academic and practical spheres. The lack of knowledge hinders the ability to draw definitive conclusions and develop evidence-based recommendations for teams in different contexts. Without a clear understanding of these mechanisms, teams may struggle to effectively implement and sustain agile practices. This underscores the need for further investigation into the factors that contribute to SAU and the development of practical strategies to support teams in their agile journey. By addressing the aforementioned limitations, this study aims to provide a more comprehensive and actionable framework towards understanding mechanisms through which human factors drive sustained agile success.”*  In the section “Management support and sustained agile usage” **(line 361 of the revised manuscript)** the following was added and modified.  **Line 377-379 of the revised manuscript:**  *“Given the significant role of the construct in providing resources, training, and a supportive culture, this study posits that MS is a critical foundational step in establishing and sustaining agile practices within teams.”*  **Line 379-383 of the revised manuscript:**  *“However, Senapathi and Srinivasan [18] were unable to quantitatively establish a statistically significant relationship between MS and agile usage from a sample of 114 agile practitioners. While this study provides a useful starting point, a larger sample size would be necessary to draw more definitive conclusions. Furthermore, while the study examined usage of practices, it did not address the nuances of SAU.”*  In the section “The individual mediating role of agile training in the impact of management support on sustained agile usage**” (line 387-388 of the revised manuscript)**, the following was added and modified.  **Line 406-409 of the revised manuscript:**  *“However, extant studies often overlook the comprehensive impact of AT on SAU, focusing primarily on how coaches help maintain practices. This study therefore aims to incorporate the various ways in which team members can access AT, beyond the traditional role of the agile coach.”*  In the section “The individual mediating role of agile mindset in the impact of management support on sustained agile usage” **(line 420-421 of the revised manuscript)**, the following was added and modified.  **Line 438 of the revised manuscript:**  *“Nevertheless, these studies show limited quantitative evidence.”*  **Line 455-456 of the revised manuscript:**  *“Furthermore, prior studies lack a strong theoretical conceptualisation of AM when examining its impact on SAU.”*  In the section “The individual mediating role of team resilience in the impact of management support on sustained agile usage” **(line 466-467 of the revised manuscript)**, the following was added and modified.  **Line 488-489 of the revised manuscript:**  *“However, the impact of TR on the specific outcome of SAU has not been probed in prior research.”*  **Line 503-504 of the revised manuscript:**  *“These findings, however, remain quantitatively unverified.”*  In the section “The serial mediation role of agile training, agile mindset and team resilience in the impact of management support and on sustained agile usage” **(line 509-510 of the revised manuscript)**, the following was added and modified.  **Line 524-525 of the revised manuscript:**  *“However, this serial effect has not been tested empirically, thus risking overlooking the nuanced and complex relationships between the variables.”*  In the section “Necessary conditions of sustained agile usage” (**line 529 of the revised manuscript)**, the following was added and modified.  **Line 552-554 of the revised manuscript:**  *“Such a limitation hinders a comprehensive understanding of what factors are indispensable in the ASD context.”* |
| 3. **Over-reliance on Older Sources**: Several of the sources cited in the review are outdated, with some being more than five years old. While classic references can be useful, there should be a stronger emphasis on recent studies to reflect the current state of research in the field. Including more up-to-date studies, particularly from the past three years, will improve the relevance of the review. | Thank you for the comment which is well-pointed out. Our study does indeed cite older sources.  However, we humbly note that the reason for more focus on choosing the classical sources is thus. To date, there has been limited quantitative research to validate the qualitative model proposed by Senapathi and Drury-Grogan (2017) on the critical success factors for sustained agile usage.  *15. Senapathi M, Drury-Grogan ML. Refining a model for sustained usage of agile methodologies. J Syst Softw. 2017;132: 298–316. doi:10.1016/j.jss.2017.07.010*  This has led to a critical paucity of the literature, stunting the literary development on the concept of SAU and its antecedents in the last seven years. Addressing this holds immense implications for future research. Our study builds on and attempts to provide a quantitative verification of these human factors at the team level, thus rekindling the discourse on agile sustainability.  Nevertheless, following your rightfully pointed out comment, we understand that citing newer sources is beneficial. we have incorporated the following studies in our new critical analysis. These were used to describe the current status of research in SAU and the broader domain of ASD. Note that the reference number of the studies are included in front.  *54. Adzgauskaite M, Tam C, Martins R. What helps Agile remote teams to be successful in developing software? Empirical evidence. Inf Softw Technol. 2025;177: 107593. doi:10.1016/j.infsof.2024.107593*  *26. Mamakou XJ. Intentions to continue using agile methods: The case of the Greek banking sector. J Syst Softw. 2023;202: 111685. doi:10.1016/j.jss.2023.111685*  *66. Meckenstock J-N. Shedding light on the dark side – A systematic literature review of the issues in agile software development methodology use. J Syst Softw. 2024;211: 111966. doi:10.1016/j.jss.2024.111966*  *68. Vishnubhotla SD, Mendes E. Examining the effect of software professionals’ personality & additional capabilities on agile teams’ climate. J Syst Softw. 2024;214: 112054. doi:10.1016/j.jss.2024.112054*  *56. Alami A, Zahedi M, Krancher O. Antecedents of psychological safety in agile software development teams. Inf Softw Technol. 2023;162: 107267. doi:10.1016/j.infsof.2023.107267*  *57. Alami A, Krancher O, Paasivaara M. The journey to technical excellence in agile software development. Inf Softw Technol. 2022;150. doi:10.1016/j.infsof.2022.106959*  *69. Tyagi S, Sibal R, Suri B. Empirically developed framework for building trust in distributed agile teams. Inf Softw Technol. 2022;145: 106828. doi:10.1016/j.infsof.2022.106828*  *30. Senapathi M, Strode D. Challenges to Sustaining Agility: An Exploratory Case Study. Proceedings of the 39th ACM/SIGAPP Symposium on Applied Computing. Avila Spain: ACM; 2024. pp. 810–817. doi:10.1145/3605098.3635926*  *55. Steegh R, Van De Voorde K, Paauwe J. Understanding how agile teams reach effectiveness: A systematic literature review to take stock and look forward. Hum Resour Manag Rev. 2024; 101056. doi:10.1016/j.hrmr.2024.101056*  *56. Strode D, Dingsøyr T, Lindsjorn Y. A teamwork effectiveness model for agile software development. Empir Softw Eng. 2022;27: 56. doi:10.1007/s10664-021-10115-0* |
| 4. **Lack of Theoretical Framework:** The literature review lacks a clear and cohesive theoretical framework. While several concepts are mentioned, it is not clear how they connect to form a theoretical foundation for the research. The authors should consider integrating a well-established framework or theory that will guide the development of their hypotheses and analysis. | Your constructive criticism is well noted. Adding a theoretical justification would be greatly helpful in establishing the connection between the variables and the study’s context, as well as help the development of hypotheses through providing a sound basis for the importance of research gaps. Furthermore, highlighting the theoretical basis would be helpful in providing justifications for the key variables.  Taking the comment into account, we have incorporated the **‘Socio-technical Systems Theory’** as a theoretical underpinning for the study. This theory is widely applied in the information systems and technology industry and highlights the importance of human factors in ensuring the success of information technology initiatives. Thus, it underpins the importance of the nature of the chosen variables, strengthening the study’s context.  The description of the theory in context of the present study is included in the section as “*The human perspective of agile teams”* **(line 281-293 in the revised manuscript)**. The section puts the theory into context of sustained agile usage.  *“The socio-technical theory of information system research [22,59] posits that any system, such as an agile team [60], comprises two interconnected subsystems: the social subsystem and the technical subsystem. The social subsystem encompasses the human elements, including team members, management, their interactions, behaviours, attitudes, team dynamics and cultural norms. The technical subsystem, on the other hand, consists of the tools, technologies, practices and processes employed within the system [61]. The two subsystems show a substantial reciprocal influence on each other, ultimately shaping the nature and direction of the holistic system [62]. However, from a sociotechnical perspective, the technical subsystem serves as a tool that empowers individuals and teams, rather than dictating their actions. This perspective emphasises humans as the active agents in shaping the technical component [63]. Therefore, successful change within a socio-technical system, such as the optimisation of agile, relies primarily on its social element [64]. Thus, this theory provides a strong theoretical foundation for assessing the human factors that influence agile adoption and sustainment.”*  Next, to provide justification for a comprehensive mediation model delineating how the human factors interact, we have incorporated the **Input-Mediator-Output-Input model of teams**.  The section reads thus **(line 335-348 of the revised manuscript):**  *“In accordance with the sociotechnical perspective [22,59], it can be argued that the human aspects within the system of an ASD team interact with each other. These interactions thereby impact the technical subsystem through critical dependencies. The Input-Mediator-Output-Input (IMOI) model of teams [68] provides a theoretical foundation to understand how these effects unfold. It illuminates that inputs, such as management activities, team training and team member attitudes, trigger the development of team processes and emergent states [27,55,57,58,69,70], such as team resilience. These processes and emergent states, in turn, gives rise to desired team outcomes, such as sustained agile usage in this context. It additionally elaborates that outcomes are further propelled by interactions between inputs, emergent states, and between inputs and emergent states. Thus, it is reasonable to posit that the factors of MS, AT, AM and TR impact SAU through dynamic interactions that have a serial impact on the same. However, a holistic investigation of these factors within a single study on agile infusion is notably absent from the literature. Due to this, a cohesive framework that addresses the mechanisms through which these factors dynamically impact SAU is still lacking.”*  In addition, the following was included in the introduction section, to provide context on how the two theories relates to the study context of human factors of sustained agile usage, while emphasizing their importance to justify the variables **(line 86-96 in the revised manuscript)**.  *“The sociotechnical theory [22] posits that the optimal performance of a system, such as an ASD team, hinges on the interplay between social and technical elements. The social subsystem is comprised of human factors like team members, management, attitudes, behaviours and interactions. These actively shape the holistic system by determining how technical tools and processes are used. This perspective, reinforced by the Input-Mediator-Output-Input (IMOI) model of teams, emphasises that interactions between such social elements lead to the development of team emergent states. In turn, these emergent states trigger team outcomes like SAU. Hence, it is evident that the efficacy of agile methodologies within teams is critically contingent upon its human factors and their interactions [6,11,23–27]. Despite this, their neglect by both scholars and practitioners [11,28,29] exacerbates challenges in sustaining agile practices within teams.”* |
| 5. **Inadequate Justification for Key Variables**: The literature review does not adequately explain why the specific variables (e.g., sustained agile usage, management support, team resilience) were selected for this study. More discussion is needed to justify the inclusion of these variables and their importance in the context of agile practices. This will help in building a stronger rationale for the research model. | We express our appreciation for the constructive comment.  We understand that the study variables’ importance has not been adequately stated.  The importance of sustaining practices in agile teams (integration, evolution and infusion) is due to the fact that realising the benefits of agile requires the repeated and correct use of methodologies and practices, as adapted per the operating context.  To make this clear in the manuscript, we have included the following **(line 199-212 of the revised manuscript)**.  *“Agile methodologies are a response to a critical need for SD teams to be flexible, adaptable and continually competitive in the midst of numerous challenges. [7–11]. However, while the initial adoption of agile methodologies is a critical first step, this is insufficient to ensure the long-term realisation of agile benefits [15,19,21,26,46,47]. Bhattacharjee [17] argues that to make the best use of any innovation or methodology, it needs to be used enduringly and persistently while being adapted to suit the operating context. It is therefore implied that contextual optimisation of the practice is crucial. Without such sustained use, the application of agile methodologies within teams would remain primitive, highlighting the importance of the concept of SAU. However, teams often encounter obstacles in their efforts towards the same [19,21,30,46], constituting a significant business problem. Hence, making a clear distinction between adoption and sustained usage of agile practices is essential to comprehensively assess the implications of the same. Thus, the current study aims to make a valuable contribution to the understanding of ASD by investigating the post-adoption or sustained stage of agile methodologies.”*  Furthermore, the significant gaps in the research on ASD and SAU also warranted the investigation of agile infusion. These gaps are described **(Line 251-279 of the revised manuscript)** in the responses to the comment 1.  Furthermore, the importance in assessing the human factors that affect SAU is that, generally, the success of agile teams, outcomes and practices is rooted in human factors. The interactions between MS, AT, AM and TR are highly relevant in ensuring a conducive social environment within the context of an agile team looking to sustain practices.  However, as you pointed out, the importance of these variables needs to be emphasized further.  Thus, we have explained this in the manuscript thus **(line 281-334 of the revised manuscript):**  *“The socio-technical theory of information system research [22,59] posits that any system, such as an agile team [60], comprises two interconnected subsystems: the social subsystem and the technical subsystem. The social subsystem encompasses the human elements, including team members, management, their interactions, behaviours, attitudes, team dynamics and cultural norms. The technical subsystem, on the other hand, consists of the tools, technologies, practices and processes employed within the system [61]. The two subsystems show a substantial reciprocal influence on each other, ultimately shaping the nature and direction of the holistic system [62]. However, from a sociotechnical perspective, the technical subsystem serves as a tool that empowers individuals and teams, rather than dictating their actions. This perspective emphasises humans as the active agents in shaping the technical component [63]. Therefore, successful change within a socio-technical system, such as the optimisation of agile, relies primarily on its social element [64]. Thus, this theory provides a strong theoretical foundation for assessing the human factors that influence agile adoption and sustainment.*  *Empirical evidence recognises the fundamental role of human factors in the success of agile practices [11,15,19,28,58,60,65,66]. The ‘Agile Manifesto’ [24] emphasises the importance of human factors over technical processes. As such, it is human-related challenges that pose significant barriers to the successful implementation and sustainment of a team’s agile practices [23,67]. Senapathi and Strode [30] elaborate that the path to agile sustainment is riddled with challenges related to the people involved and their lack of readiness to embrace agile values. They cite the lack of agile mindsets, leadership support, and unconducive team dynamics as factors. This aligns with Denning's [16] assertion that true agility requires a cultural shift, going beyond the mere adoption of practices to embrace agile values and behaviours. To be precise, teams are required to ‘be agile, rather than do agile’. Therefore, insufficient scholarly and practitioner focus on such human factors significantly inhibit the optimisation of agile methodologies, However, despite the importance of human factors within the context, research providing strong evidence for the role of such factors in SAU is still in infancy. Hence, applying a socio-technical lens to the study of SAU, this research aims to investigate how specific human factors contribute to successful agile sustainment.*  *In their seminal work, Senapathi and Drury-Rogan [15] qualitatively introduced and refined a foundational framework for the critical success factors of organisational SAU. In this model, they underlined the pivotal roles of sociological influences. MS, by way of actively facilitating the implementation of agile through providing resources, training, creating a supportive culture and empowering teams, was found to be instrumental in fostering SAU. Furthermore, they elucidated the vital role of the AM. They asserted that its inherent characteristics such as the enthusiasm to learn, willingness to collaborate, proactive and independent initiative, and customer-centricity were central to ensuring agile assimilation in teams. Likewise, the importance of AT and mentoring of team members to this end was demonstrated to be significant. It was recognised that the imparting of technical and business knowledge pertaining to agile methodologies while facilitating agile team dynamics through such training is crucial for SAU. However, the study failed to address the perspective of the ASD team, instead investigating the relationships on the organisational level. Besides, subsequent research has failed to build upon this seminal study with recent empirical verifications. Furthermore, limited quantitative analysis has been carried out with regards to the impacts of the variables on SAU. Thus, the importance of the MS, AT and AM for team level SAU remain unresolved and requires further empirical investigation.*  *Moreover, researchers such as Cheng et al. [31] emphasise the importance of the ability of SD teams to effectively adapt to change, overcome challenges, and maintain their performance in order to achieve agile success. This coincides with the concept of TR which denotes the ability of teams to weather change and adversity with minimal functional detriment [29,55,65]. Since SAU requires ongoing change, it is reasonable to posit that TR is a prominent factor in the success of the same. However, the lack of prior research on TR in the context of SAU presents a significant opportunity to explore its impact on agile infusion. Accordingly, given their strong theoretical and empirical foundation in ASD literature, this study selected MS, AT, AM, and TR as key human factors to investigate their impact on SAU.”* |

- **Methodology**

| **Reviewers’ Comment** | **Response to the Comment** |
| --- | --- |
| 1. **Sample Size Calculation**: The authors used a robust approach to calculate the minimum required sample size using the inverse square root method and the guidelines by Hair et al. However, it would be beneficial if the authors provided more detail on how the anticipated path coefficients (0.11-0.2) were determined. Were these values based on previous studies or preliminary data? Providing this context would strengthen the justification for the sample size calculation. | We are profoundly grateful for your constructive insight and agree that providing this context would strengthen the justification for the sample size calculation.  The estimated path coefficients we used were based on the study by Senapathi and Srinivasan, who probed a comparable conceptual background relating to agile usage.  *18. Senapathi M, Srinivasan A. An empirical investigation of the factors affecting agile usage. Proceedings of the 18th International Conference on Evaluation and Assessment in Software Engineering. New York, NY, USA: Association for Computing Machinery; 2014. pp. 1–10. doi:10.1145/2601248.2601253*  Furthermore, heeding your valuable suggestion, we have removed the sentence “Based on anticipated path…approximately 155 was calculated.” (line 433-435 on the original manuscript) and included the following **(line 569-573 in the revised manuscript).**  *“A statistical power of 80% and a significance level of 5% was employed. Anticipated path coefficients of 0.11 to 0.2 were determined based on the study by Senapathi and Srinivasan [3], which explored a similar conceptual framework related to agile usage. Thus, a sample size of approximately 155 was calculated for the current study.”* |
| 2. **Sampling Approach**: The stratified multi-stage cluster sampling approach is appropriate for ensuring representation across different company sizes, but the authors could provide additional details on how the strata were determined. For example, was there any justification for the chosen employee size range (10-200 for SMEs, over 200 for large companies)? Further, clarity on how agile team members were randomly selected from within each stratum would enhance transparency. | The comment is noted with due appreciation and thanks.  The stratification of companies into SMEs (10-200 employees) and LSEs (over 200 employees) was based on the Sri Lankan national policy framework for small and medium enterprise development (service enterprises), as outlined by the Sri Lankan Ministry of Industries and Commerce. This classification is widely recognised and used in Sri Lanka. To incorporate this additional information, the description of the sampling process was modified and expanded as shown **(line 577-582 in the revised manuscript).**  “*The list of companies was stratified based on employee size into small to medium-sized enterprises (10-200 employees) and large-scale enterprises (over 200 employees). The classification aligns with the national policy framework for small and medium enterprise development put forward by the Sri Lankan Ministry of Industries and Commerce [93]. This framework defines small to medium-sized enterprises in the service sector as those employing fewer than 200 people*”  Furthermore, to ensure clarity on how agile team members were randomly selected from within each stratum, the following description was included **(line 580-585 in the revised manuscript).**  *“Companies, serving as clusters, were then randomly selected from each stratum. Within each selected company, a random sample of agile teams was chosen based on assigned team numbers. Finally, eligible ASD employees were randomly selected from each team using their employee numbers to form the final study sample. This multi-stage cluster sampling approach allowed for efficient and cost-effective data collection while ensuring adequate representation from different segments of the population.”* |
| 3. **Pilot Testing and Questionnaire Validation**: The authors conducted a pilot test on 45 agile software development professionals and consulted an agile expert for content validation. While this strengthens the reliability of the instrument, more details on the specific changes made after the pilot test would be useful. What kind of feedback was received, and how did it influence the final version of the questionnaire? | We are extremely grateful for your thoughtful commentary.  In response, we have included the below paragraph section detailing the changes incorporated to the questionnaire following the pilot test **(line 590-593 in the revised manuscript).**  *“The pilot test and expert feedback revealed respondent concerns about survey length, ambiguous language, item redundancy and repetitiveness. Ambiguous items were rephrased to enhance clarity. Redundant and repetitive items were either removed or merged to streamline the survey. Thus, the overall length of the survey was reduced from 84 to 51, retaining the most critical items to minimise respondent fatigue.”* |
| 4. **Response Exclusion**: The exclusion of 21 responses due to "suspicious response patterns" is noted. However, it would be beneficial if the authors provided more detail on the criteria used to identify these suspicious patterns. Were these cases of straight-lining or other forms of non-engaged responses? Clearly stating the exclusion criteria would help in understanding how data quality was maintained. | We sincerely appreciate the helpful comment on improving the clarity of our data quality assurance measures.  Incorporating the feedback, the following section (line 452-454 of the original manuscript) was revised and expanded, as indicated **(line 602-605 in the revised manuscript).**  “*While no missing data were reported, 21 responses were excluded due to a lack of variability within each response, indicating cases of straight lining. These responses were identified by a standard deviation of less than 0.25 within a single response*.” |
| 5. **Demographic Breakdown**: The demographic breakdown of the respondents is well-presented, including gender, experience, company size, and team size. However, it may be valuable to provide a discussion on the potential impact of gender imbalance (77.75% male respondents) on the generalizability of the findings. Are there any implications of this skewed distribution for interpreting the results? | This comment regarding the potential effect of gender imbalance on the generalisability of findings is very well-placed and well-received, with thanks. We acknowledge that this is a valuable point of discussion.  However, gender (male vs. female) did not show a significant differential impact on SAU, when employed as a control variable (significance level = 0.314 > 0.05). Hence, we did not include the same as a variable in study.  Furthermore, we humbly note that the Global Gender Gap Report 2024 by the World Economic Forum indicates that women represent only 37.64% of the ICT workforce in Sri Lanka and 28.2% globally in STEM fields. The actual proportion of women in software development teams, both globally and in Sri Lanka, would be lower. Therefore, the gender imbalance observed in our study aligns with broader industry trends.  To address this in the context of our current study, we have thus included the following sentence **(line 608-611 in the revised manuscript).**  “*This gender imbalance aligns with broader industry trends. The Global Gender Gap Report 2024 by the World Economic Forum [94] indicates that women represent only 37.64% of the overall ICT workforce in Sri Lanka and 28.2% globally in STEM fields.”* |

- **Results**

| **Reviewers’ Comment** | **Response to the Comment** |
| --- | --- |
| 1. **Detailed Descriptions of Constructs:** While the mean scores are provided for each construct, a brief interpretation of these values in terms of their implications for the study context would add depth. Discussing the significance of the mean scores being above average could offer insights into the current state of agile practices among the study population. | We sincerely appreciate you bringing this to our attention. Incorporating this into the manuscript will indeed offer insight into the current state of the agile practices within the industry.  Thus, the following was added **(line 789-792 in the revised manuscript).**  *“This indicates that the teams, on average, exhibit strong levels of SAU, MS, AT, AM and TR. Thus, it implies that the teams potentially have adopted a strong foundation for agile principles and practices, a promising start to long term benefit realisation.”* |
| 2. **Visual Representation**: Consider including visual representations (such as graphs or charts) alongside the descriptive statistics. This could enhance reader engagement and provide a clearer snapshot of the data distributions and correlations. | Duly noted with appreciation.  Taking this into account, we have included a boxplot as Fig 2 (*figure caption: Fig 2. Data distribution (Source: Created by authors using the Datatab application based on collected data*)) detailing the measures of central tendency and dispersion, to provide a clearer context of the data **(line 800 in the revised manuscript).**    The below sentence was added to contextualise the graph **(line 794-795 in the revised manuscript).**  *“The boxplot in Figure 2 visualises the sample’s descriptive statistics, providing a snapshot of the data’s distribution.”*  The figure numbers of the subsequent figures were adjusted accordingly. |
| 3. **Measurement Model Assessment Details:** The authors reference established guidelines for evaluating construct validity but could benefit from including more details on the specific tests conducted. For instance, explicitly stating the thresholds used for reliability and validity could strengthen the rigor of the analysis. | We gratefully acknowledge your valuable feedback and constructive insight.  We very humbly note that we have already incorporated details on specific tests and thresholds of construct validity in the sections describing the assessment of each specific criterion (item-level reliability, internal consistency reliability, convergent validity and discriminant validity).  For instance, the following was already previously included on line 642-646 in the original manuscript, regarding the assessment of item-level reliability **(line 820-827 of the revised manuscript)**. Note that the citations have been updated in the revised manuscript.  “*Item-level reliability was assessed using the outer loadings of each measurement item. Following the guidelines of Hair et al. [92], the conventional minimum threshold for an outer loading stands at 0.5, with a desirable value of 0.708. Items with loadings between 0.40 and 0.70 were contemplated for removal only if doing so resulted in a significant improvement in overall reliability and validity [34]. However, in this study, no such improvement was observed. Moreover, the reported lower-order outer loadings ranged from 0.634 to 0.942, exceeding the minimum threshold and demonstrating satisfactory item-level reliability (Table 2).*”  Furthermore, regarding the assessment of internal consistency reliability, the following was stated on line 649-655 of the original manuscript **(line 827-835 of the revised manuscript)**.  “*Next, the internal consistency reliability of each lower-order construct was probed using Cronbach’s alpha (CA) and composite reliability (CR) values [95], both of which should ideally fall between 0.70 – 0.95 [34,35]. However, in an instance when other construct validity measures yield satisfactory values, values between 0.60 – 0.70 are deemed acceptable [34,92]. It should also be noted that criticisms of Cronbach’s alpha as being conservative and less accurate include its tendency to understate the reliability [35]. In comparison, CR tends to perform better with multidimensional constructs*. *With CA values ranging from 0.619 to 0.932, and CR values ranging from 0.814 to 0.948, the measures showed adequate internal consistency reliability at first-order level.”*  Next, details on the assessment of convergent validity were mentioned thus on 657-660 in the original manuscript **(line 835-840 of the revised manuscript):**  “*Proceeding to the assessment of convergent validity, the average variance extracted (AVE) was utilised for the same. According to Hair et al. [35], a minimum AVE of 0.50 is considered acceptable, indicating that the construct explains at least half of the variance in its indicators*.”  Next, the assessment of the discriminant validity has been detailed thus on line 662-666 of the original manuscript **(line 842-846 of the revised manuscript):**  “*Next, the discriminant validity of the measures was verified using the Heterotrait-Monotrait ratio (HTMT ratio) [96], and cross-loadings. An item, Cap02, exhibited ambiguous cross loadings with another construct and was thus removed. As presented in Table 3, all items fell within the acceptable threshold of less than 0.90 [34,35], establishing discriminant validity at lower-order with a maximum value of 0.889.”*  Please note that the higher order assessment used the same thresholds, as specified in the manuscript **(line 812 to 817 of the revised manuscript).**  *“Given the presence of the second-order variables of AM and TR, the study used the disjoint two stage approach of measurement model assessment [107,114,120]. This approach involved initially assessing the measurement model at the first-order level, followed by an evaluation at the second-order level. The construct validity was assessed at each level, using the indicator-level reliability, internal consistency reliability, convergent validity, and discriminant validity [39,40,118].”*  However, the brief referencing of the established guidelines (line 630-633 of the original manuscript) and their detailed explanations are separated by other text. This could potentially disrupt the flow of the argument, hinder readability and cause confusion. Therefore, we have removed the brief reference in the revised manuscript, presenting the detailed explanation upfront. |
| 4. **Justification of Reflective Model:** While the authors state that all constructs were modeled as reflective, it might help to briefly justify this decision. A discussion of why reflective measurement was appropriate in this context, as opposed to formative measurement, would clarify the methodological choices. | Thank you very much for your keen and detailed commentary. As you have rightly pointed out, justifying the decision would definitely add more clarity and validity.  The reflective measurement model was deemed appropriate following the application of Coltman et al.’s framework for determining the reflective vs. formative nature of constructs.  *119. Coltman T, Devinney TM, Midgley DF, Venaik S. Formative versus reflective measurement models: Two applications of formative measurement. J Bus Res. 2008;61: 1250–1262. doi:10.1016/j.jbusres.2008.01.013*  Additionally, we have modelled all constructs as reflective based on how they were modelled in key studies that were important in conceptualising the variables, developing hypotheses as well as in forming and refining the study’s measurement scales.  We have incorporated this justification into the text thus **(line 804-812 in the revised manuscript).**  “*All constructs were modelled as reflective, in accordance with Coltman et al’s [119] framework for determining the reflective or formative nature of a construct. The underlying latent variables of MS, AT, AM, TR and SAU exist independently of their measures, with variations in each construct causing variations in their item responses. Specifically, the items were observed to be manifestations of the underlying constructs, sharing a common theme and being interchangeable without altering their constructs’ conceptual domains. Aligning with this, key studies that informed the conceptualisation, hypothesis development and measurement of the variables in the current research have also employed reflective models [11,18,81].*” |

- **Discussion**

| **Reviewers’ Comment** | **Response to the Comment** |
| --- | --- |
| 1. The discussion section is well-presented. | Thank you very much for your positive feedback on the discussion section. It is a great honour to receive such esteemed validation. |
| 2. Authors should expand theoretical and practical implications. | Your insightful comment is greatly appreciated. We acknowledge that further detail on theoretical and practical implications would enhance the paper's contribution. Considering your valuable input, we have incorporated the following revisions into the new manuscript.   - Regarding the theoretical implications:   We have strengthened the discussion on the implications of the use of advanced methods such as NCA and IPMA. The modified section on the cIPMA application reads thus **(line 1113-1125 of the revised manuscript):**  *“First, this study offers a significant methodological contribution by pioneering the combined use of NCA and IPMA to complement PLS-SEM (cIPMA) within the context of ASD. Thus, it provides evidence for the ability of such techniques to address critical research gaps within ASD research. Through the incorporation of IPMA, the study successfully builds an empirical reference point for how the performance of teams can be assessed. Furthermore, while studies in agile research have identified the necessity of certain factors for SAU, this study is among the first to empirically test these necessity hypotheses. By employing NCA, the study provides concrete evidence for the role of MS, AT, AM, and TR as essential conditions for agile success. Through the fundamental understanding of the relationships thus established by incorporating both sufficiency and necessity logic, the study lays the groundwork for future ASD studies to explore these concepts with even greater nuance. Additionally, the advanced methodological implementation of cIPMA can serve as a reference for other researchers seeking to conduct similar investigations and contribute to the ongoing development of the field.”*  To emphasise the fact that the current study incorporates TR into the discourse on SAU, we have added the following sentence on **line 1129-1130 of the revised manuscript**.  *“It extends the discourse on SAU as well as its antecedents by incorporating TR, a factor previously unexplored in the context of agile infusion.”*  We have emphasised the importance underlying the holistic understanding of the variables, taking the socio-technical systems theory into account. Thus, we show that our study furthers theoretical understanding by modifying the following **(line 1130-1133 of revised manuscript).**  *“Using the socio-technical systems theory as a basis, it amalgamates the largely fragmented research on these key constructs to map a more holistic illustration of how the relationships unfold within a team. This cements evidence of their importance in the arena of ASD.”*  Next, we have incorporated a section detailing the study’s contribution to the post adoptive phase of agile teams **(line 1141-1148 of the revised manuscript).**  *“Third, our study responds to a timely call for a re-evaluation of the current understanding of ASD. Given the significant research gap in understanding the post-adoption phase of ASD, this study emerges at a crucial juncture, poised to stimulate a much-needed academic discourse. Providing a comprehensive exploration of distinct factors influencing the post adoption phase, and elucidating the complex interplay between these factors, this research lays the foundation for future studies in this emerging field. Furthermore, it addresses a critical paucity of literature addressing SAU at the team level, increasing the applicability of the research to the granular level of the ASD team.”*  Next, we have emphasised the importance of the quantitative verification in the factors affecting SAU by adding the following **(line 1154-1156 of revised manuscript).**  *“Furthermore, this quantitative verification of factors affecting SAU will pave the way for future research centred on SAU, stimulating further discourse and development in this area”.*  Next, we have emphasised the importance and implication of addressing the human factors in agile development thus **(line 1157-1165 of the revised manuscript):**  *“Fifth, while the technical aspects of agile methodologies are often emphasised, the human factors that underpin agile success are equally important. This study contributes to the field by examining the critical role of understudied human factors in sustaining agile usage. By drawing on sociotechnical theory, it operates on a strong theoretical base to explore how the interplay between social factors (MS, AT, AM, TR) impacts the success of the teams’ agile practices. Thereby, this study offers a valuable empirical basis for how teams can manipulate these towards developing targeted strategies to enhance agility. Furthermore, it makes a profound contribution to the ongoing discourse on the human dimensions of agile, demonstrating the complexity of the same.”*   - Practical implications   The practical implications of the use of cIPMA as a planning tool within teams is emphasised by adding the following **(line 1171-1179 of the revised manuscript):**  *“Similarly, the incorporation of formal analyses such as cIPMA can offer teams a systematic approach to identify and address performance gaps, enhancing the overall effectiveness of their agile practices. The study demonstrates the practical applicability of cIPMA as a tool for strategic assessment and planning within teams. In other words, the applicability of cIPMA as a powerful tool within teams to identify risks and bottlenecks, and plan resource allocation accordingly is illustrated. This will, in turn guarantee improved and insightful decision making, having a favourable impact on proactive risk mitigation. Thereby, such thorough applications will be monumental in ensuring that the team is receptive to the benefits promised by agile methods, securing competitive advantage.”*  The following section was added to enhance readability, flow and clarity **(line 1180-1181 of the revised manuscript):**  *“Therefore, further practical implications and resulting recommendations of the study are hereby expounded separately for both managers and team members.”*  The implications of the findings on the managers of ASD teams were further expanded **(line 1183-1185 of the revised manuscript).**  *“However, it is also observed that management support is lacking in teams. Thus, this highlights the implication that more effort is required on the part of the top management to facilitate the processes within teams.”* |
| 3. Conclusions and limitations should be separate. | Thank you very much for bringing this to our attention.  The content of the section ‘Conclusion, limitations and future research’ has been split into two sections as *‘Conclusion’* **(line 1214 in the revised manuscript)** and ‘Limitations and future research’ **(line 1225 in the revised manuscript)**. |

**Additional Reviewers’ Comments**

| **Reviewers’ Comment** | **Response to the Comment** |
| --- | --- |
| 1. Avoid using abbreviations in research questions. | Thank you very much for bringing this to our attention. We have replaced the abbreviations with their full forms, as indicated in the research **(line 156-166 in the revised manuscript).**  “*RQ1 – Does management support have a significant positive impact on sustained agile usage?*  *RQ2 – Do agile training, agile mindset, and team resilience individually mediate the relationship between management support and sustained agile usage, and how important is each in the sequence?*  *RQ3 – Do agile training, agile mindset, and team resilience serially mediate the relationship between management support and sustained agile usage?*  *RQ4 – How does management support, agile training, agile mindset, and team resilience, perform compared to their importance in fostering sustained agile usage?*  *RQ5 – Are management support, agile training, agile mindset and team resilience* *necessary but not sufficient conditions for sustained agile usage*?” |
| 2. Minor changes are also recommended in the document. | We thank you for you valuable feedback and suggestions. We are very grateful for your effort towards improving the manuscript through your keen attention to detail. The mansuscript was largely improved when addressing the review comments. |
| 3. Do not use abbreviations in headings. | Well noted, thank you.  This has been adjusted in all main headings and sub-headings of the revised manuscript. |
| 4. Do not use abbreviations in hypotheses. | Humble apologies for the error.  We have adjusted the needful, incorporating the full forms of the variables.  *“H_1_: Management support has a significant positive impact on sustained agile usage”* **(line 386 of the revised manuscript).**  *“H_2_: Agile training positively and significantly mediates the impact of management support on sustained agile usage”* **(line 418-419 of the revised manuscript).**  *“H_3_: The agile mindset positively and significantly mediates the impact of management support on sustained agile usage”* **(line 464-465 of the revised manuscript).**  *“H_4_: Team resilience positively and significantly mediates the impact of management support on sustained agile usage”* **(line 507-508 of the revised manuscript).**  *“H_5_: Agile training, agile mindset, and team resilience positively and significantly serially mediate the impact of management support on sustained agile usage*” **(line 527-528 of the revised manuscript).**  *“H_6_: Management support is a necessary condition for sustained agile usage”* **(line 557 of the revised manuscript)**.  *“H_7_: Agile training is a necessary condition for sustained agile usage”* **(line 558 of the revised manuscript)**.  *“H_8_: Agile mindset is a necessary condition for sustained agile usage”* **(line 559 of the revised manuscript).**  *“H_9_: Team resilience is a necessary condition for sustained agile usage”* **(line 558 of the revised manuscript).** |
| 5. H2, H3, and H4 are required to be rewritten or hypothesized again. | We appreciate you bringing this to our attention.  Please note that hypotheses H_2_ – H_5_ have been rewritten, incorporating the word ‘impact’ instead of ‘relationship’. This would be more appropriate in the current study’s context, given the study background and analytical technique.  The changes incorporated are as follows (as detailed in the response to the previous comment):  “*H_2_: Agile training positively and significantly mediates the impact of management support on sustained agile usage*.” **(line 418-419 of the revised manuscript).**  “*H_3_: The agile mindset positively and significantly mediates the impact of management support on sustained agile usage*.” **(line 464-465 of the revised manuscript).**  “*H_4_: Team resilience positively and significantly mediates the impact of management support on sustained agile usage.”* **(line 507-508 of the revised manuscript).**  “*H_5_: Agile training, agile mindset, and team resilience positively and significantly serially mediate the impact of management support on sustained agile usage.”* **(line 527-528 of the revised manuscript).** |
| 6. A stratified multi-stage cluster sampling approach was used and described. However, a description of cluster sampling is missing. | We sincerely apologise for the failure to integrate the explanation.  To rectify this, and give a more comprehensive account of cluster sampling, we have included a more detailed description of how the cluster sampling process was carried out, in the methods section.  The section is as follows **(line 582-587 in the revised manuscript):**  *“Companies, serving as clusters, were then randomly selected from each stratum. Within each selected company, a random sample of agile teams was chosen based on assigned team numbers. Finally, eligible ASD employees were randomly selected from each team using their employee numbers to form the final study sample. This multi-stage cluster sampling approach allowed for efficient and cost-effective data collection while ensuring adequate representation from different segments of the population”.* |
| 7. An explanation should follow each table. Moreover, Table 3 is described along with Table 2, and the remaining description of Table 2 is given after Table 3. | Thank you very much for bringing this to our attention. We respectfully apologise for the confusion.  However, we would like to humbly clarify the reason we refer to the contents of Table 2 multiple times throughout the section, even after Table 3.  The measurement model assessment for this study was conducted and explained in two sequential steps to accommodate the higher-order constructs of AM and TR, as per the disjoint two stage approach. At each step, reliability and validity measures were assessed. For conciseness, as well as to avoid result fragmentation and repetition, all the results for indicator-level reliability, internal consistency reliability, convergent validity from both steps were combined and presented in Table 2. Table 3 details the results of the discriminant validity at lower order. Thus, after the lower order assessment, we refer to table two again when describing the higher order assessment. Furthermore, the outer weight assessment obtained in the measurement model is a preliminary analysis for IPMA. To avoid repeating items, we incorporated all this into a single table (Table 2).  Nevertheless, we acknowledge that this may be confusing and reduce readability. Thus, in line with the feedback, we have incorporated the following changes.  First, the section “The aforementioned assessments were conducted…denote the results obtained at higher order.” (line 633-637 in the original manuscript) was replaced by the following (**line 812-817 in the revised manuscript).**  “*Given the presence of the second-order variables of AM and TR, the study used the disjoint two stage approach of measurement model assessment [81,88,94]. This approach involved initially assessing the measurement model at the first-order level, followed by an evaluation at the second-order level. The construct validity was assessed at each level, using the indicator-level reliability, internal consistency reliability, convergent validity, and discriminant validity [39,40,118].”*  Next, we separated the details of the higher order constructs (AM and TR) from table 2. We included these details in a new table as Table 4 **(line 856 of the revised manuscript)** captioned *“Table 4. Measurement model assessment – higher order (Source: Authors’ calculation)”.* The subsequent table numbers were adjusted accordingly.  Next, we have separated the first explanation of the Table 2 from that of Table 3 by splitting into two distinct paragraphs. In the revised manuscript, the explanation for Table 2 is given on **line 818-840 of the revised manuscript**, while that of Table 3 lies on **line 842-846 of the revised manuscript**.  Furthermore, the following section was added, to make the changes clearer **(line 818-819 in the revised manuscript).**  “*Table 2 presents the item-level, internal consistency reliability, and convergent validity results for first-order constructs*.”  Kindly note that, following the above revision, the first mention of Table 2 was moved from line 640 in the original manuscript to **line 818 in the revised manuscript.** Therefore, in accordance with PLOS ONE's guidelines, we have placed Table 2 on **line 841 in the revised manuscript**, directly after the paragraph in which it is now first cited.  Next, we have separated the outer weight details (both lower and higher order) from Table 2 and 4. Then, we have included these details into a new table as Table 7 **(line 929 of the revised manuscript)** captioned *“Table 7. Outer weight assessment for IPMA – lower and higher order (Source: Authors’ calculation)”.*  The subsequent table numbers were adjusted accordingly. |
