# Supplementary material for: Does management support drive sustained agile usage? a serial mediation model and cIPMA perspective
Source: PLoS One. 2025 Feb 5;20(2):e0316538. doi: 10.1371/journal.pone.0316538 (PMC11798488; doi:10.1371/journal.pone.0316538)
Supplement: Attachment — Submitted filename: Response to Reviewers.docx [file pone.0316538.s005.docx]

**Journal Requirements**

| **Comments** | **Response to the Comment** |
| --- | --- |
| Please review your reference list to ensure that it is complete and correct. If you have cited papers that have been retracted, please include the rationale for doing so in the manuscript text, or remove these references and replace them with relevant current references. Any changes to the reference list should be mentioned in the rebuttal letter that accompanies your revised manuscript. If you need to cite a retracted article, indicate the article’s retracted status in the References list and also include a citation and full reference for the retraction notice. | We sincerely thank you for bringing this to our attention.  To address this concern, the reference list was completely reorganised.  First, the following citation (line 1260-1261 of the originally revised manuscript) was removed, and the subsequent references were numbered accordingly.  5. Rathor S, Batra D, Xia W, Zhang M. What Constitutes Software Development Agility? 2016.  Next, the reference list was adjusted to reflect the cited order. Thus, the reference below, previously cited under citation number 126 (line 1591-1593 of the originally revised manuscript), was numbered as 93 **(line 1516-1518 in the newly revised manuscript)**.  93. Wisenthige K. Research Design. In: Saliya CA, editor. Social Research Methodology and Publishing Results: A Guide to Non-Native English Speakers. IGI Global Scientific Publishing; 2023. p. 74–93. doi:10.4018/978-1-6684-6859-3.ch006  Then, each reference was individually updated and made consistent to include complete reference details in the correct formats. For example, issue numbers and page numbers were included where available. The full forms of the journal names were included for clarity.  The newly reviewed and adjusted reference list can be found on **line 1236-1620 in the newly revised manuscript**. |

**Additional Editor’s Comments**

| **Editor’s Comments** | **Response to the Comment** |
| --- | --- |
| Table 6: R2 and F2 is mentioned; the 2 should be in superscript. | We are grateful for your keen attention to detail.  We have adjusted the formatting as requested in both the caption and in the table content **(line 911 in the newly revised manuscript)**. |
| Table 7 and Table 9, and their supporting discussion do not add substantial value to the IPMA (Importance-Performance Map Analysis) section. If they do not provide new insights or actionable data beyond what is already discussed. The manuscript is already too detailed and it is my suggestion that only IPMA with table 8 is enough, which shows construct-level results. | Your insightful comment is greatly appreciated. We acknowledge that Table 7 and Table 9 do not offer substantial additional value.  In response, the tables 7 and 9 were removed for a more concise yet comprehensive discussion. The subsequent table numbers were adjusted accordingly.    The subheadings ‘Importance-performance map analysis at construct level’ (line 931 of the originally revised manuscript) and ‘Importance-performance map analysis at indicator level’ (line 950 of the originally revised manuscript) were removed. Their contents were merged for better conciseness **(line 929-950 in the newly revised manuscript)**.  To improve the flow of the newly merged section, the discussion of MS on the construct level was moved from line 937-939 of the originally revised manuscript, to **line 939-942 in the newly revised manuscript**. This ensures a better continuation of the construct’s discussion on indicator level.  The fully adjusted section regarding the IPMA on the construct level, on **line 929-949 in the newly revised manuscript,** reads thus:  *“Fig 4 and Table 7 provide a comprehensive representation of the IPMA results at construct level. The analysis revealed that TR in the upper-right quadrant exhibits the highest relative importance of 0.500, indicating its pivotal role in driving SAU. However, with an above-average performance of 75.214, there is a low potential for further increase. A one-unit increase in TR from 75.214 to 76.214 (as shown in Table 7) can lead to a significant increase of 0.500 points in the performance of SAU. Relative to TR, a one-unit increase of AM in the upper-left quadrant from 79.373 to 80.373 leads to an increase of 0.391 points in SAU’s performance, which, while very slightly below average, is still substantial. On the other hand, AT in the lower-left quadrant exhibits trifling importance (0.224), and slightly below average performance in the furtherance of SAU (73.492). Thus, increasing the performance of AT does not lead to as large an increase in SAU. Relative to the other constructs, MS in the lower-right quadrant shows a considerable importance of 0.492, yet its actual performance at 69.963 falls short of the average performance of constructs. This reveals an area of critical concern for practitioners.”*  The previous detailed description of the IPMA at the indicator level has been omitted. To still emphasize the importance of scrutinizing the construct MS (as its of low performance yet high importance), the following has been included **(line 947-949 in the newly revised manuscript)**:  *“The IPMA of MS at the indicator level (Fig 5) corroborates the findings at the construct level. The underperformance of MS is attributable to and reflected in deficiencies across all five of its indicators. This further underpins the vital need for attention to this construct.”* |
